# Special Hospitals

**Published:** 1860-09

**Authors:** 


*Special Hospitals.*—A movement having been set on foot to found a Special Hospital for the treatment of stone and diseases of the urinary organs, in London, a number of the most prominent physicians of that city, among whom are Brodie, South, Latham, Watson, etc., have published an address in the Lancet objecting to this, and expressing their opinion on special hospitals generally, as follows:

"The practice is injurious, First, because in the maintainance of numerous small establishments the funds designed for the direct relief of the sick poor are wasted in the useless multiplication of expensive buildings, salaries and hospital appliances, and in the custom of constantly advertising to attract public attention.

"Secondly, because the public is led to believe that particular classes of diseases can be more successfully treated in the small special institutions than in the general hospitals—an assumption directly contrary to evidence; the fact being that the resources of the general hospitals are in every respect superior to those of the special institutions alluded to.

"Thirdly, because it is essential for the interests of the public, with a view to the efficient education of students preparing for the practice of the medical profession, that all forms of disease should, as far as possible, be collected in the general hospitals to which medical schools are attached."

